# Manufacturing Magic and Computational Creativity

**DOI:** 10.3389/fpsyg.2016.00855

**Published:** 2016-06-10

**Authors:** Howard Williams, Peter W. McOwan

**Affiliations:** School of Electronic Engineering and Computer Science, Queen Mary University of LondonLondon, UK

**Keywords:** magic, optimization, AI cards, jigsaw computer, computational creativity

## Abstract

This paper describes techniques in computational creativity, blending mathematical modeling and psychological insight, to generate new magic tricks. The details of an explicit computational framework capable of creating new magic tricks are summarized, and evaluated against a range of contemporary theories about what constitutes a creative system. To allow further development of the proposed system we situate this approach to the generation of magic in the wider context of other areas of application in computational creativity in performance arts. We show how approaches in these domains could be incorporated to enhance future magic generation systems, and critically review possible future applications of such magic generating computers.

## 1. Introduction

This review focusses on the theoretical context of a conceptual framework, proposed by Williams and McOwan (2014), for the creation and optimization of magic tricks, based on observations of psychological phenomena that inform computational search and optimization techniques. An overview of how computational systems can, and do, contribute to human creative endeavors in many fields will be provided, along with suggestions for how work in these diverse areas can potentially further inform automated magic trick creation. Additionally, the degree to which computational systems can be viewed as creative entities in their own right will be discussed. A brief overview of psychological research that may be useful for new automated magic design systems is also given, leading to a discussion of the areas of future work, and potential new tricks, that lie ahead.

The framework detailed in Williams and McOwan (2014) outlines a set of conceptual tools that allow for a systematic approach to understanding and subsequently designing magic tricks, with the creative assistance of various computational systems.

Magic, as a performance art, has been around for thousands of years; Christopher and Christopher (1996) provides an excellent history. The performance of any magic trick is vital to its success. However, the design of the trick itself—the set of methods, and physical objects (props and gimmicks) that must be deployed for a strong effect in the spectator (a seemingly magical event)—is equally important. The perception of a trick by a spectator can be influenced by both of these factors, and by the spectator themselves. For a trick to have a strong magical effect there must be a cohesive interaction between these three elements: a trick's designer, performer, and spectator.

As Ortiz (1994) notes, a magical effect occurs in the mind of a spectator. For example, Lamont and Wiseman (1999) describe one of the most commonly performed magical effects, the *vanish*: most people will have seen someone make a coin vanish from their hand. This is one of a number of basic magical effects that magicians are able to achieve; others include, *appearance*: an object seems to impossibly come into existence; *transposition*: an object miraculously moves in space; *transformation*: an object is changed to another form; *restoration*: a previously destroyed object is reconstituted; *penetration*: the impenetrable is breached. An object, in this context, can be a physical object, or a piece of information—for example, a playing card, or a spectator's date of birth. Essentially, an effect is an event that the observer perceives as being something outside of the normal physical rules of the world; see Lamont and Wiseman (1999). Further useful descriptions of the basic effects achieved by magicians is provided in Smith et al. (2016).

Often, effects will be achieved by **influencing perception**, via a sleight of hand, misdirection, or a device utilized by the magician. Whatever particular approach is used to create a specific effect, the mechanism is traditionally referred to as the method; Ortiz (1994) provides detailed discussions of methods for magic tricks.

KEY CONCEPT 1 **Influencing perception**

The human perceptual system can be manipulated and affected by both external physical, and internal psychological, processes to produce magical effects, and there exist optimal configurations of these factors that give rise to better magic experiences.

Magic tricks have, throughout history, been designed by ingenious human inventors, such as Robert Harbin, U.F. Grant, Fred Braue, Alex Elmsley, and many more; knowledge of fundamental techniques is passed between magicians and designers, under a widely observed code of secrecy forbidding the dissemination of information to the uninitiated. These human trick designers tend to evaluate the quality of their creations through informal analysis and intuition (which they are exceptionally good at). See Rissanen et al. (2013) and Rissanen et al. (2014), for qualitative studies that describe some of these types of approaches.

However, sometimes a trick's design (and subsequent evaluation of that design) will present problems that are challenging for even the most ingenious of humans using informal methods. The dissection and rigorous scientific analysis of the various factors necessary for a trick to be successful would surely provide benefits to a trick designer similar to those reaped by creative humans in fields more historically closely aligned with science and engineering, such as architecture and software design. Further, and critical to this review, a formal analysis of magic tricks allows for the possibility of a computational system to be imagined that can aid human beings with the task of designing magic tricks. Fortunately, magic has been a subject of scientific interest for some time—the application of psychological theories to magic was investigated by Triplett (1900) over a hundred years ago. It is difficult to determine the earliest rigorous scientific study of magic, though Jastrow (1897) produced investigations in this area in 1897. Interestingly, both these studies describe magic's efficacy as being closely related to its narrative powers: heightening the psychological impact of the magical effect by building to its climax, using various psychological methods along the way; for example, establishing the magician as the wielder of extraordinary powers by way of a number of small magical effects. These types of concepts can be difficult to naively formalize from a computational perspective; specific data must be generated in a form that can be meaningfully manipulated by a computer, with measurable outputs that can be further subject to scientific evaluation.

There have been a number of attempts to create a set of scientifically sound principles to describe the techniques involved in magic and conjuring. Binet (1894) used a chronophotographic gun (enabling the rapid recording of sequential photographic frames) to investigate sleights of hand used by magicians, revealing previously unknown perceptual mechanisms. Nardi (1984) approached magic tricks from a sociological and social psychological perspective, analysing the similarities and differences between a magic performance and interactions during normal life—the work shows how a magician is able to construct an alternate version of reality by bracketing off parts of the performance, and setting up various visible and concealed “tracks" of events, in order to control and undermine people's expectations, and their normal rational view of the world. Hyman (1989) analyzed the psychology of deception, providing a historical overview, along with suggested categorizations and examples of the various types of deception. Later, Wiseman (1996) laid out some foundational works toward a psychological theory of deception, before Lamont and Wiseman (1999) made an effort, supported by interviews with leading magicians, to explain the theoretical and psychological underpinnings of conjuring tricks, outlining a number of rules and fundamental techniques that can be used for effective performance.

Critically, Kuhn et al. (2008) started work toward postulating a general science of magic, a way of investigating magical effects from a scientific perspective, categorizing and formalizing the various physical and psychological processes involved. This kind of effort provides concrete and measurable theories as to the general nature of human perception and cognition as it relates to magic tricks. Further, Rensink and Kuhn (2015a) have summarized how magic tricks themselves have been evaluated scientifically. They remark that the use of appropriately controlled experiments to rigorously determine, for example, that a particular effect does or does not exist, may enable the underlying mechanisms, psychological or otherwise, to be described. An example of this type of detailed scientific investigation into magic is how Parris et al. (2009) used neuro-imaging techniques to show that while perceiving a magic trick, certain brain regions associated with the detection of conflict and the implementation of cognitive control were more highly activated in the left hemisphere—further, viewing of magic tricks caused greater activations in these regions than viewings of surprising events (the former differentiated from magical events by the imposition of a magic condition: that a cause-effect relationship is violated); this suggests that the brain regions identified play a special role in causality processing, and constitute an element of the neurobiology of disbelief.

There are opposing views as to the usefulness of studying magic from a scientific perspective, or framing a theory of magic in scientific terms: Lamont et al. (2010) suggest that it is in itself an illusion that a science of magic exists, arguing that the link between theories of conjuring, and scientific theories of psychology, have been exaggerated. More recently, Lamont (2015) argues against the possibility of discovering the kind of natural taxonomies of magic tricks, see Kuhn et al. (2014), or methods, see Rensink and Kuhn (2015a), that have been proposed as worthy of investigation. Part of Lamont's argument appears to rely on the idea that different effects may be realized using a similar method. Rensink and Kuhn (2015b) have responded by pointing out that this type of small variation over sets of items has not hindered other scientific endeavors of this type, for example the categorization of animals in biology. It seems that while varying magical effects are possible from similar methodological bases, this does not rule out the possibility of stratifying the various effects and methods, including the components of the variations, themselves. In some senses, varying effects can be produced through the use of psychological lenses that are applied by a magician via these subtle variations in the method—while the method remains the same up to a point, the final effect is determined with a small but crucial change that alters the spectator's perception of the events. This does not rule out the possibility of describing the various lenses, though may increase the difficulty of the challenge. This will surely be an ongoing process—for example, new living organisms are routinely observed and codified.

As mentioned, for an automated trick design system to be built, such as that described by Williams and McOwan (2014), it is necessary that not only are the components of the trick understood in a formal sense, but also that the system is designed in a way that enables the outputs to be objectively evaluated with respect to the perceptual and cognitive phenomena associated with a spectator's experience of a given trick. Regarding the former, recent work by Smith et al. (2016) provides an example of how a magic trick may be dissected into logical states and actions, showing how a trick's success depends on the construction of a type of logical impossibility for the spectator, achieved through the use of parallel event sequences (one for the performer, and one for the spectator). Considering the latter, formalized psychological research can provide reliable data about particular mental phenomena — data sets gathered using these methods can be integrated into computational systems for **modeling perception**. The data may be related to either the spectator or performer of a magic trick, or in certain cases both, and can also be used by a human trick designer to inform the design process.

KEY CONCEPT 2 **Modeling perception**

Human perceptual systems can be modeled mathematically and/or computationally in order to optimize magical effects.

Designers can of course choose to deploy computers to aid the trick design process in much the same way that writers may use word processing software, or film makers video editing software—for example, stage illusionist designers use computer-aided design (CAD) packages to design large on-stage props. However, this application of computers is superficial to the core of the effects, being used instead to enhance the design aesthetics, or improve ease of fabrication and construction.

Williams and McOwan (2014) have presented and investigated a conceptual framework, for the design, optimization, and evaluation of magic tricks that explicitly integrates the various elements of magic discussed in a rigorous, formalized way, including the addition of a computational component that is intended to provide solutions to computationally hard problems that are unavailable to human designers. This computational component is realizable only through the assumption, and application of, some form of a science of magic. The role of the computer in their proposed framework is seen to be fundamental, allowing trick designers to move a large degree of responsibility for a trick's core design to the software, with the intentions of improving the resultant effect for a spectator, and easing the designer's task in various ways. Further, the framework allows magic tricks themselves to be studied from a scientific perspective. This type of system shows the computer's primary role to be one of **assisting design** in meaningful ways. Configuring a computer to perform this kind of role is neither straight forward, nor fully realizable. Creative computers, able to specify their own parameters in order to produce entirely novel categories of artefacts for human consumption, are still arguably some way off. Computers that assist the design process in a given domain (e.g., for **manufacturing magic**) in significant ways, some of which appear to mimic aspects and outputs of human creativity, are, however, currently feasible.

KEY CONCEPT 3 **Assisting design**

Computational systems can take on areas of responsibility in the design process of certain types of magic trick.

KEY CONCEPT 4 **Manufacturing magic**

Configuring computational systems with empirically determined psychological data, within a flexible and extensible conceptual framework, can successfully produce novel magic trick designs.

## 2. The battle for computational creativity

Building computational systems to assist with various tasks has a rich and varied history. Early efforts in artificial intelligence focussed on problem solving systems that, configured with a formalized knowledge of a particular domain, would process logical operations, via a general inference engine, over the parameters of the domain, in an effort to find solutions to given problems; see Newell et al. (1959). The idea was to create a generalized, universal, problem solver, capable of solving any problem that could be stated in formal terms. The system had successes with relatively simple tasks, but ran up against the difficulty of combinatorial explosion: the various ways in which a logical system could be combined, even with only a small number of components, became intractably large.

Knowledge based systems, built with sophisticated inference engines, generally termed expert systems due to the extensive input from human domain experts, provide queryable access to well formed knowledge bases. An early system used for medical diagnosis, developed at Stanford University, see Buchanan and Shortliffe (1984), while never used operationally, often performed better than its human counterparts. Such systems, once configured, can provide intelligent answers to varied queries, often more accurately and quickly than their human counterparts due to their vast memory banks, and speed of processing. While impressive, such systems are not seen as creative, in the sense that they are not designed to output novelties, rather to locate previously acquired knowledge efficiently.

While the framework detailed in Williams and McOwan (2014) bears similarities to aspects of expert systems, domain experts are consulted (magicians) and their knowledge formalized and operated on by computers, the goals, and therefore the implementations of the systems differ. Expert systems aim to provide accurate and detailed answers to queries based on existing knowledge bases, while, arguably, the framework aims to provide a way to build systems that output artefacts that contribute to these existing knowledge bases.

Computational creativity is the field that addresses the challenge of building creative computational systems that output novel artefacts. The idea, if not implementation, of creative computers, distinct from problem solvers, or expert knowledge systems, reaches back to the very beginnings of computing. However, this notion of machines as creative entities in their own right was intially met by some with scepticism. Ada Lovelace, described as the world's first computer programmer due to her work detailing uses of Charles Babbage's Analytical Engine (the first general purpose computing machine, never built), see Babbage (1889), understood that this new machine would be capable of creating new works of music of any degree of complexity, but she also believed that the credit for these must be given to the engineer that configures the machine, not the machine itself:

The Analytical Engine has no pretensions whatever to originate anything. It can do whatever we know how to order it to perform. It can follow analysis; but it has no power of anticipating any analytical relations or truths (Lovelace, 1842).

From this perspective, the systems described in Williams and McOwan (2014) are no more or no less creative than the people that created them, and would be incapable, in present or future form, of generating tricks unimagined by their authors.

Alan Turing, the originator of the modern general purpose computer, and deep thinker on the topic of the possibility of machine intelligence, disagreed with Lovelace on this matter:

A variant of Lady Lovelace's objection states that a machine can “never do anything really new.” This may be parried for a moment with the saw, “There is nothing new under the sun.” Who can be certain that “original work” that he has done was not simply the growth of the seed planted in him by teaching, or the effect of following well-known general principles (Turing, 1950).

Viewed in this way, the systems in Williams and McOwan (2014) may be categorized as formalized creative entities, generating novel artefacts in a similar, if hugely simplified, manner to their human counter parts.

At the crux of the argument lies the problem of understanding what it means to perform a creative act—a difficult problem. Boden (1998) supplies a neat summary of what lies at the source of creative thinking:

Creativity is not a special “faculty,” nor a psychological property confined to a tiny elite. Rather, it is a feature of human intelligence in general. It is grounded in everyday capacities such as the association of ideas, reminding, perception, analogical thinking, searching a structured problem-space, and reflective self-criticism.

Following work in Newell et al. (1963), Boden identifies the criteria that the output of creative systems, human or computational, must be *novel* (to the system itself) and *useful* (evaluated as such). For example, a new, or variant of an existing, magic trick—Boden (1998) describes three different type of creativity that humans, or computer systems, may engage in:

1. Combinational—the novel combination of two or more familiar ideas.
2. Exploratory—the generation of novel ideas by the exploration of structured conceptual spaces.
3. Transformational—the transformation of one or more dimensions of a structured conceptual space, enabling the acquisition of previously unavailable new ideas.

Interestingly, although transformational creativity is the most radical of the three, and likely to result in the most unexpected new ideas, it is in fact exploratory creativity that the majority of creative people are engaged in, as Boden (1998) describes:

Many human beings—including (for example) most professional scientists, artists, and jazz-musicians—make a justly respected living out of exploratory creativity. That is, they inherit an accepted style of thinking from their culture, and then search it, and perhaps superficially tweak it, to explore its contents, boundaries, and potential.

We can add magic trick designers to Boden's list of creative human beings.

From a computational perspective, creative artefacts have many parameters governing their perceived quality. Combining these in an optimal way can be a difficult task for a human, due to the large number of combinations available. The design of some artefacts, or elements thereof, can fundamentally be seen as a search problem—exploratory creativity. Mitchell (1998) explains that search problems fall into three categories: locating targets in a search space (pattern matching), optimizing a cost function (optimization), or path planning. A cost function (or objective function, or fitness function) is a measure of the quality of the solution found by an optimization algorithm. Solving a search problem, for a computer, entails traversing either physical data stores of some kind, or virtual spaces as defined by a mathematical function. Systems built using cost functions will often have soft and hard constraints imposed upon their outputs—hard constraints being those that define invalid solutions in a search space, while soft constraints are those aspects of the solution that should ideally be maximized, or minimized, as appropriate.

In a search space, there may be valid solutions that are not in fact the best possible solution in the entire space; these are local optima. The best possible solutions in the search space are referred to as the global optima. Optimization is the effort to find parameter values, within a set of constraints, that produce an optimal solution to a particular problem. Some optimization methods are able to guarantee returning global optima, though may not be able to do so in a practical amount of time, depending on the particular domain.

Much work in the field of computational creativity uses search and optimization techniques to explore controlled problem domains (parameter spaces) in search of novel and optimal artefacts (pieces of music, paintings, magic tricks, etc). There has been an effort in Wiggins G.A. (2006), Wiggins G. (2006) to formalize Boden's notions of creativity in to a computational framework, noting the similarities between exploratory creativity and many computational search methods, in order to better explore the conceptual underpinnings and try to lay out a way forward that could encompass the automation of creative acts. Colton et al. (2011) build on these ideas and present a computational creativity theory that contains both a descriptive model of creative acts (that they term FACE), and a descriptive model of the impact that computationally creative acts may have (termed IDEA). For example, they built systems to generate visual art, then evaluated the impact these works had on people that knew they had been generated computationally.

In Williams and McOwan (2014), the authors present systems that would appear to be engaged in just this kind of exploratory creativity; systems that trawl search spaces looking for novel configurations of data that represent new magic tricks. On the surface, these systems may appear to be engaging in creative acts; their outputs are certainly novel. To further explore this notion, it is necessary to place these systems within a wider context. Colton and Wiggins (2012) summarizes seminal works in the field of computational creativity, and provides a brief history of the topic, along with a working definition of what it is:

The philosophy, science and engineering of computational systems which, by taking on particular responsibilities, exhibit behaviors that unbiased observers would deem to be creative.

They contrast such systems with those produced in the HCI (human-computer interaction) field, see Shneiderman (2007), that assist human beings to generate creative work; for example Photoshop (visual art), Max/MSP (new media), AutoCAD (engineering/architectural/product design), Cubase (music), Eclipse (software development) etc. The main difference, they argue, is that computationally creative systems take on responsibilities for the creation of artefacts that HCI systems generally do not. They also introduce the idea of an unbiased assessor to fairly evaluate the outputs of computationally creative systems. They note the readiness with which human beings attribute creativity to the programmer and not the machine. Interestingly, they see computational creativity as moving away from a problem solving paradigm, the most common approach in the wider AI field, toward:

…an *artefact generation* paradigm, where the automation of an intelligent task is seen as an opportunity to produce something of cultural value (Colton and Wiggins, 2012).

Creative computational systems have been implemented in many fields. A detailed history is given in Cardoso et al. (2009). The systems developed in each area are often radically different to one another in the approach they take to generating novel and useful outputs, using different kinds of data structures, and applying different algorithms to the problems at hand, tailoring each to the specific conceptual space. Magic, as a craft, synthesizes many different types of creative endeavor to produce a final trick.

What follows is a short review of some of the important advances made in computational creativity, seen to be the natural domain into which the framework under discussion falls, rather than a comprehensive overview of the state of the art, along with a brief discussion of how these areas may feed in to further work on producing autonomous systems for magic trick creation.

### 2.1. Design science

Cross (2001), provides a discussion of the history of attempts to “scientise" design from the 1960's on, and makes the distinction between design as a discipline, as a way for humans to engage in designing successfully, and design as a science in which the design methods are formalized, with a hope that works of art and design may be produced in an objective manner. Various applications have sprung from this systematic approach to design, including automating information sytems, see Hevner and Chatterjee (2010), architectural layouts, see Michalek et al. (2002), and combinational circuit design, see Coello et al. (2000). The framework of Williams and McOwan (2014) makes some first steps at formalizing an automated design methodology as applied specifically to the automated generation of artefacts for use in magic tricks. The use of empirical psychological data is seen as critical to the creation of successful designs for magic. Further developments and refinements in this area should certainly be fruitful, in particular for the design of the physical items involved in much magic (props and gimmicks).

### 2.2. Language, stories, and poetry

Poetry, perhaps the most specifically human of all the arts, has been subjected to a computational approach for its generation. Natural language, particularly of the ambiguous kind typically used in poetry, and magic performances, is very difficult terrain for a computer. Gervás developed a computer system, ASPERA, to compose formal Spanish poetry, see Gervás (2000). Dìaz-Agudo et al. (2002) describes a similar system, COLIBRI. These expert systems (a system that relies heavily on the formalization of knowledge from domain experts) use case based reasoning (the generation of solutions to problems based on known solutions to similar problems) to generate poetic versions of inputted text by querying a database of previously written poems. Oliveira (2009) provides a more comprehensive overview of the various approaches to automatic poetry generation.

Sardonicus, described in Veale and Hao (2007), is also a case based system; it constructs a database of similes for adjectives from data on the internet, which is then used by a system named Aristotle to suggest new metaphors for provided descriptive goals.

The MINSTREL system, described in Turner (1994), generates short stories of reasonable quality (given their origin); the system is based on the idea of separating out, and formalizing, the goals of the characters in a story from the narrative goals of the author. An intelligent search procedure is performed on a database of known previous answers to the problems that meeting these goals throws up, resulting in novel stories.

These types of systems can plausibly be deployed for generating and integrating narratives for use in magic tricks. For example, in Williams and McOwan (2014), the tricks described each use a narrative to maximize the impact of the effect; automating and/or optimizing the narrative generation process is an unexplored area, and one likely to throw light on the psychological processes at work in human observer's of magic tricks with relation to language and its ambiguities and nuances.

### 2.3. Comedy

It is well known that magic and humor go naturally together; magicians are, however, wary of over using humor during their routines for fear of reducing the impact of the magical effect. This is an obvious area for optimization—using computational systems to generate humorous sections of a trick may allow for the optimization of both the jokes themselves, and for the timing and frequency of their deployment, and how this may impact the overall effect.

Humor is often attempted via computers: the JAPE (Joke Analysis and Production Engine) system, see Binsted et al. (1997), was an early successful development, capable of generating puns that young children found humorous by analysing and formalizing the structure of certain types of jokes and finding a way to score new candidate jokes for meaning and humor. The JAPE system is also interesting because the authors used empirical methods to evaluate the quality of the creative artefacts that it produced; the jokes it produced were consistently rated, by children, on a par with human created jokes of a similar kind.

A system that incorporates some kind of formal understanding of how much, or what kind, of humor is best deployed in a magic trick can be imagined.

### 2.4. Visual art

Perhaps the most successful and controversial creative system developed is the Painting Fool from Colton Colton (2012), an AI project with the aim of one day being taken seriously as an autonomously creative visual artist. The Painting Fool is a hybrid system, using various techniques to automatically generate the elements of a picture. The project also investigates the sociological implications of the idea of computational artists, and the impact the created artefacts have with the general public. This aspect of the work, its impact with the public, is of particular relevance to magic. As Williams and McOwan (2014) note, the introduction of technological objects into a magic performance runs the risk of removing some of the magicians narrative tools and performance devices necessary to the successful creation of a sense of wonder for the spectator. Revealing that the trick has, at least in part, been created by a computer, increases this risk. There are many factors to consider, and avenues to research.

The creation of visual artefacts has an obvious link to magic, which often has a visual effect at its core. The significant successes creating visual art computationally provide a rich source of knowledge about how imagery and its perception by a spectator can be manipulated toward certain desired effects—a natural domain for magic.

### 2.5. Music

Rigorously defining the various facets of the perception of music, from a psychological perspective, is difficult. The perceptual and cognitive processes at work while a magic trick is observed are similarly nebulous. Analysing computational systems that produce music in a creative way, or otherwise, can throw light on ways in which systems may be structured for the production of magic tricks. Methods for evaluating the eventual artefacts produced by each of these types of system is critical to them having any hope of success (creative autonomy), and is an ongoing field of research.

Horner and Goldberg (1991), McIntyre (1994), Papadopoulos and Wiggins (1998), and Phon-Amnuaisuk et al. (1999) all describe Genetic Algorithms (GAs) as the core process in a software system engineered to compose music. These systems all computationally evaluate the quality (or, in GA terms, the fitness) of the outputs during the iterative process of creation. Similar approaches that instead use a human assessor, known as Interactive GAs (IGAs), have also been developed by, among others, Horowitz (1994), Ralley (1995), and Biles (1994). The main limitation of this interactive approach is that a human must assess the outputs of the system at each stage of the evolutionary process, which is inefficient. Spector and Alpern (1995) describes Genetic Programming (GP; the programming code itself is evolved) methods for the creation of musical phrases intended to respond to other phrases in jazz music. Johanson and Poli (1998) describes GP-Music, another GP system, that also uses automatic fitness (quality) assessors. Iliopoulos et al. (2002) describe an evolutionary system capable of generating musical motifs in polyphonic passages (more than one note at any given time) to order. Chuan and Chew (2007) describe a hybrid system (with assessments and input from both computer systems and humans) that focusses on creating accompaniments in a specific style.

The idea of building computer systems that first learn a model of a particular conceptual space, for example music, and then having them alter the model to generate new artefacts, is the subject of a large theoretical effort by Wiggins, see Wiggins (2012), whose work relies on Baars' Global Workspace Theory, see Baars (1993), as a conceptual framework, the statistical modeling of musical perception pioneered by Pearce (2005), and ideas from Shannon's Information Theory; see Shannon (1948).

Being able to effectively encode the structure of a magic tricks performance, both the qualitative and quantitative elements, so that it can be represented in a form suitable for manipulation to generate new variants would be challenging. Unlike music where large data sets, all comprising musical notes, exist, and can be learned to extract models for music, no such common coding or annotated data sets exists for performance. Music is encoded note by note, but it is not clear what the resolution of the representation of a magic performance encoding would need to be to prove effective and be able to discriminate between performance nuances. This could prove an interesting area for future work.

## 3. Conjuring creativity: future work

In the previous section we described several areas where findings from other diverse areas of research in computational creativity could provide fertile avenues to further enhance the creation of new magic. In this final section we look at domains in psychology and magic where appropriate computational creativity frameworks could be applied.

### 3.1. Exploiting computational psychology

Magic has a wide psychological scope, that exploits many facets of human perceptual and cognitive systems; Rensink and Kuhn (2015a) provide an overview of many principles and parameters that have been investigated in this area. Here, we highlight some areas of psychological phenomena that may be most relevant for use in computational systems configured to generate magical effects.

Our brain's expectations of the perceived world plays a large part in what we consciously experience, even when it differs from the actual reality of the situation. Triplett (1900) and later Kuhn and Land (2006) developed and studied a vanishing ball illusion whereby a ball is tossed in the air several times by a magician, before it appears to suddenly vanish in mid-air during the final (simulated) toss. Latterly, Kuhn and Rensink (2016) show how studying magical effects, specifically the vanishing ball illusion, can shine a light on cognitive processes at work during their viewing; for example, predictive visual processes, and the effect of long term knowledge, and exemplars from the immediate past, on perception. Work done by Griffiths and Tenenbaum (2006) highlights the apparent similarities between probabilistic statistical reasoning, and the ways in which the human brain operates—this type of mathematical modeling of expectation by the brain should have a natural application in computational magic trick optimization processes.

A magician's success in secretly changing or moving objects during a performance may depend largely on their ability to surreptitiously move their spectator's attention around the scene they have created for their trick, be it a stage, or a deck of cards in their hands. Attention is seen in Desimone and Duncan (1995) as the process that enables our brains to selectively filter large amounts of incoming perceptual information, and to make sense of and manipulate our environment in advantageous ways. This sophisticated and complex process can also lead to perceptual errors, such as important visual information being ignored in certain circumstances. The computational modeling of visual attention is a large topic area, see Itti and Koch (2001); incorporating specific instances of these types of models for use in magic trick optimization would appear feasible.

Change Blindness, the inability to detect large changes in visual scenes without consciously attending to them, is a relatively large topic area that has been critically assessed in Simons (2000). Rensink et al. (1997) shows how the human perceptual system does not form complete detailed representations of visual scenes, and that attention is the crucial element required to perceive changes in an environment under normal viewing conditions. To determine this experimentally, Rensink uses a *flicker paradigm*: people are shown two images, A and A', with a blank screen interleaved between them for a short period. A and A' are identical, except for some small, or large, changes made by the researchers to A'. People struggle to identify even very large changes made to the scenes depicted in A'. Interestingly, Verma and McOwan (2005) has shown how computational models of saliency perception, coupled with computational optimization techniques, allow for the semi-automated generation of visual stimuli that predict change detection performance. The study shows that low level saliency is a reasonable predictor of change detection performance in comparison with high level measures (e.g., mouse-click densities). It should be possible to harness these predictive models for computational magic trick optimization.

Attention can be manipulated by perhaps the most important tool available to a magician: misdirection—in one sense, the art of moving an observer's attention away from a point of interest, allowing the magician to perform a move of some kind (slipping something into a pocket, swapping a card, etc). Misdirection is not well understood. Kuhn and Martinez (2011) provide an overview of the current thinking, from both magicians and scientists, on the role of misdirection in magic, and explanations of the basic principles at work. Kuhn et al. (2014) provide an extensive taxonomy of misdirection in magic, based on the perceptual and cognitive mechanisms involved.

There is work to be done to formalize and fully understand all the perceptual and cognitive mechanisms at work in misdirection, though there exists much that is of interest to those wishing to understand misdirection from both a scientific and magical perspective. Formalizing this knowledge computationally would provide a great deal of predictive power for generating new magical effects.

### 3.2. Conjuring up new tricks

In stage magic, the perceptual effects of shading on perceived depth or shape form an arsenal of methods to fool audiences into seeing as normal and innocent configurations that in fact conceal the method. Barnhart (2010) describes how magicians routinely exploit gestalt principles in these types of stage magic. Often, in stage magic that involves a human assistant, the assistant's body is worked into a position that is physically possible, but unexpected. This is the basis of a number of classical stage illusions. Evaluating the range of positions a body can take with a reasonable degree of comfort and having these rated by observers on the basis of plausibility would provide the basic data for later optimization.

Similarly the use of, and optimization of, relevant optical illusions suggests itself; for example, the use of beveled sides to obscure the actual depth of a trick's base box, or the use of appropriate geometric shading to make large spaces look smaller, could be measured and encoded. Human designers currently use software packages to model the three dimensional problems inherent in this type of stage trick design. The addition of a more sophisticated approach that allows for the optimization of the various design parameters based on empirical knowledge of the envelope of subjects perception of “normality” appears to be a natural avenue for further research.

Marrying this type of psychological insight to modern computational power also suggests the possibility of large scale tricks on social media platforms. The sheer volume of information currently available on the internet provides a ready-made source of psychological data. Social network theory, see Wasserman and Faust (1994) and Barabási (2002), is increasingly well understood, from a computational, psychological, and sociological perspective; combining these observations with magical techniques that involve confederates and third parties could offer many opportunities for new magic. For example, using social media platforms on which confederates secretly reside, in conjunction with predictive computational models based on network theory could allow for the implementation of a magic trick in which a spectator's seemingly free choice (e.g., a particular website they choose to visit at a certain time) could be magically revealed to have been previously known.

This type of computational modeling, in which the outcome of a psychological process is predicted and optimized, can also be imagined being applied to lower level, even unconscious, systems. Close up magic, that often relies on particular attributes of the human visual system, for example through the modeling of misdirection or sensory illusions, is a good example. There is a body of research already available on the science of misdirection, see Kuhn et al. (2014) and Kuhn and Martinez (2011), and illusions, see Robinson (1998), that could be utilized and built upon. An as yet unexplored prospect is a workable computational model of misdirection that would enable the conception and optimization of a large number of new tricks. This could possibly be approached by constructing a model of visual gaze, and a model of distraction events, allowing various tests to be performed to verify the model, and subsequently allow the prediction of new tricks based on the reliable manipulation of a spectator's attention. Beth and Ekroll (2015) provide a study discussing the importance of timing in close up magic; it is precisely this type of data that could be incorporated into a concrete computational model of aspects of magical effects. Also, at a more macro level, work by Van de Cruys et al. (2015) exploring seemingly unconscious inferences about the state of the world by the brain could be built into such systems.

Similarly, we can view magic tricks as an exercise in undermining a spectator's expectations—for example, a performer picks up a ball and closes their fist around it, only to later open the same fist, revealing an empty hand. It could prove useful to explore the human brain's processing of the expectations of events, and coupling these observations with probabilistic graphical methods in computer science, see Koller and Friedman (2009), for example Bayesian Networks, to both predict optimal physical motions/properties for use in tricks, and to test our understanding of the particular psychological processes at work during their viewing.

### 3.3. New scientific knowledge

The scientific study of magic is now an active field of research, see Kuhn et al. (2008). There appear to be ways to use magic as a psychological probe, to help understand the operation of the human brain: for example, Rensink and Kuhn (2015a) proposes an extensive framework for using magic to study the mind.

There may be useful research to be conducted using aspects of the framework described in Williams and McOwan (2014) in order to study perceptual and cognitive processes by designing specific magical effects to probe particular perceptual phenomena. Analysing the efficacy, or otherwise, of such effects may illustrate underlying psychological processes—for example: expectation (seeing what we expect to see, even if it isn't there); false memories; or various attention based processes.

### 3.4. New interfaces informed by magic

Tognazzini (1993) elucidates how magical principles are at work in computer user interfaces—the user does not see the reality of the underlying computer, rather a form of user illusion that the interface presents to them.

User interface design, particularly in the field of medical devices, demands precision, simplicity, and clarity. Making mistakes, both perceptual and cognitive, is a normal part of everyday human activity, but for human beings operating in a medical context making these types of errors could have serious consequences, see Kohn et al. (2000): medical errors cost lives. There have been efforts to engineer better, safer, medical device interfaces, that go some way to reducing the possibility for human error; see Zhang et al. (2003). These have often been based on adapting user interface design methods from other fields. Informatics has recently been used to improve processes and outcomes in the healthcare of cancer, see Hesse et al. (2016).

Applying an optimization process such as that presented in Williams and McOwan (2014) could prove useful in this area of user interface design. Breaking an interface down into both psychological and physical components should allow the application of a similar design and optimization process used for magic tricks, but instead of maximizing the level of deception achieved, the number of errors produced by an interface could be minimized.

## 4. Conclusion

This paper has reviewed the development of the study of magic as a scientific field, and the arguments for and against the utility of such studies. The various psychological phenomena that contribute to an effective magic performance need to be considered if a computational system is to be able to mimic such activity. The challenges in this are as noted significant and it is necessary, if an explicit computational model is required, to abstract certain tractable elements of magical theory into a workable computational encoding.

Following this reductionist approach, a specific case of the use of computers as computational design aids for magic trick designers was presented and discussed—see Williams and McOwan (2014)—within the context of various definitions of creativity. The creativity at work during a performance, in the magician's varied and subtle interactions with members of the audience, is currently, though not necessarily, beyond the scope of such systems.

The framework developed, which optimizes over both physical and psychological constraints, was then considered within the wider context of computational creativity systems developed in other performance domains. This comparison allows the suggestion of other elements that could be incorporated to contribute to future magic design systems. The fundamental creativity of such computational systems, including those presented in Williams and McOwan (2014), was critiqued, leading to the proposal that humans remain the main creative force behind the novel outputs generated.

Finally, the case for the utility of using such simplified magic frameworks as probes to study the information processing in the human brain is made, and areas of potential future applications, making use of explicit computational models, capable of predicting observer performance are described.

## Author contributions

All authors listed, have made substantial, direct and intellectual contribution to the work, and approved it for publication.

## Funding

We thank the Engineering and Physical Sciences Research Council (EPSRC).

### Conflict of interest statement

The authors declare that the research was conducted in the absence of any commercial or financial relationships that could be construed as a potential conflict of interest.
